# mPneumonia, an Innovation for Diagnosing and Treating Childhood Pneumonia in Low-Resource Settings: A Feasibility, Usability and Acceptability Study in Ghana

**DOI:** 10.1371/journal.pone.0165201

**Published:** 2016-10-27

**Authors:** Amy Sarah Ginsburg, Charlotte Tawiah Agyemang, Gwen Ambler, Jaclyn Delarosa, Waylon Brunette, Shahar Levari, Clarice Larson, Mitch Sundt, Sam Newton, Gaetano Borriello, Richard Anderson

**Affiliations:** 1 Save the Children, Seattle, Washington, United States of America; 2 Kintampo Health Research Centre, Kintampo, Ghana; 3 PATH, Seattle, Washington, United States of America; 4 Department of Computer Science and Engineering, University of Washington, Seattle, Washington, United States of America; Centre Hospitalier Universitaire Vaudois, FRANCE

## Abstract

Pneumonia is the leading cause of infectious disease mortality in children. Currently, health care providers (HCPs) are trained to use World Health Organization Integrated Management of Childhood Illness (IMCI) paper-based protocols and manually assess respiratory rate to diagnose pneumonia in low-resource settings (LRS). However, this approach of relying on clinical signs alone has proven problematic. Hypoxemia, a diagnostic indicator of pneumonia severity associated with an increased risk of death, is not assessed because pulse oximetry is often not available in LRS. To improve HCPs’ ability to diagnose, classify, and manage pneumonia and other childhood illnesses, “mPneumonia” was developed. mPneumonia is a mobile health application that integrates a digital version of the IMCI algorithm with a software-based breath counter and a pulse oximeter. A design-stage qualitative pilot study was conducted to assess feasibility, usability, and acceptability of mPneumonia in six health centers and five community-based health planning and services centers in Ghana. Nine health administrators, 30 HCPs, and 30 caregivers were interviewed. Transcribed interview audio recordings were coded and analyzed for common themes. Health administrators reported mPneumonia would be feasible to implement with approval and buy-in from national and regional decision makers. HCPs felt using the mPneumonia application would be feasible to integrate into their work with the potential to improve accurate patient care. They reported it was “easy to use” and provided confidence in diagnosis and treatment recommendations. HCPs and caregivers viewed the pulse oximeter and breath counter favorably. Challenges included electricity requirements for charging and the time needed to complete the application. Some caregivers saw mPneumonia as a sign of modernity, increasing their trust in the care received. Other caregivers were hesitant or confused about the new technology. Overall, this technology was valued by users and is a promising innovation for improving quality of care in frontline health facilities.

## Introduction

Pneumonia is the leading infectious cause of childhood mortality worldwide. Estimates suggest that 935,000 children less than five years of age died from pneumonia in 2013, accounting for 15 percent of child deaths globally.[1] Challenging to identify, especially in low-resource settings (LRS), pneumonia often goes undiagnosed and untreated until the child is severely ill or is improperly managed by health care providers (HCPs).[2] Pneumonia-related deaths in children under five can be preventable with appropriate diagnosis, treatment and management procedures, but strategies to implement existing methods specifically tailored to LRS are needed.

Currently, paper-based protocols employing World Health Organization (WHO) Integrated Management of Childhood Illness (IMCI) and integrated Community Case Management (iCCM) algorithms are used to diagnose pneumonia, and rely on a HCP’s ability to visually observe the child’s breathing and manually count the respiratory rate, typically for one minute.[3–11] Fast-breathing is under recognized, and measuring a child’s respiratory rate accurately can be difficult in a fast-breathing, moving child, and studies have demonstrated that algorithms based only on respiratory rate do not appropriately identify children with pneumonia.[2, 12–14] Clinical assessments alone are not enough to appropriately establish the severity of the disease or the risk of complications. Pulse oximetry is a rapid, noninvasive way to assess oxygen saturation and detect hypoxemia, a diagnostic indicator of disease severity often associated with an increased risk of mortality.[15] Improving access to oxygen and pulse oximetry has been shown to reduce the risk of childhood pneumonia death by up to 35% in a high-burden setting.[16] However, hypoxemia is not routinely evaluated in LRS because pulse oximetry is often unavailable, especially in frontline health facilities where the majority of children are first evaluated for pneumonia.[17] Furthermore, in spite of it being a critical component in the management of severe pneumonia and the WHO encouraging its use, pulse oximetry is not included in the current IMCI algorithm.

Studies have shown that mobile health (mHealth)-based applications and algorithms can facilitate HCP adherence to diagnostic and treatment guidelines.[18–22] To improve HCPs’ ability to diagnose, classify, and manage childhood pneumonia, we developed “mPneumonia,” an innovative and user-friendly mobile health application using Android technology that integrates a digital version of the IMCI algorithm with a software-based breath counter and a pulse oximeter. mPneumonia is designed to be a free, open-source platform easily adapted for various countries and/or contexts. The software can integrate with other Open Data Kit (ODK)-based tools on any Android device and does not require an internet connection to operate. The software-based breath counter was designed to help HCPs more accurately count a child’s breaths and a device driver was developed for a pulse oximeter to detect hypoxemia by following step-by-step instructions. The integrated pulse oximeter is FDA-approved for medical grade use and is competitively priced. This application provided a user-friendly diagnostic and management tool and decision support system for childhood pneumonia and other childhood illnesses. Additional details about the development, field testing, and features of mPneumonia are described elsewhere.[23] In Ghana, where HCPs are currently trained to use the paper-based IMCI guidelines for diagnosing childhood illnesses, we conducted a design-stage qualitative pilot study to understand the feasibility, usability and acceptability of the mPneumonia application in frontline health facilities.

## Methods

Following initial field testing in Ghana and subsequent optimization of the mPneumonia application, we undertook a design-stage qualitative pilot study based in ground theory methods from July to September 2014 among end-users in 11 sub-district health facilities in Kintampo North and South Districts, predominantly rural areas in the Brong-Ahafo region of Ghana. The study sites included six health centers (50% of the health centers in the districts) and five community-based health planning and services (CHPS) centers (17% of the CHPS in the districts). Some of the participating CHPS centers had limited to no electricity. The objectives included determining the organizational and technical feasibility of introducing mPneumonia in frontline health clinics in Ghana, assessing the usability of mPneumonia when used by HCPs in these facilities, and understanding the acceptability of mPneumonia by HCPs and caregivers of children (Table 1). Of note, the design and development process took into consideration local health policies, health system structures, and resource availability, which are especially important in LRS. Activities were conducted in accordance with the consolidated criteria for reporting qualitative research (COREQ) (refer to S1 Table).[24]

**Table 1:** Definitions of Feasibility, Usability, and Acceptability.

Term	Definition in context of mPneumonia
Feasibility	Organizational feasibility is the structural factors that influence introduction, include the administrative infrastructure of the health system and the operational capabilities of the health facilities. Technical feasibility is the perceived capabilities and potential skills of the HCPs in the health network as well as the infrastructure requirements of the individual facility level.
Usability	Usability is the design factors that affect the user experience of operating the application’s device and navigating the application for its intended purpose.
Acceptability	Acceptability by HCPs is the factors that affect their willingness to use the application during patient interactions. Acceptability by caregivers is the factors that influence their willingness to have the application used with their children.

In order to inform the organizational and technical feasibility of introducing the mPneumonia application, district health administrators who directly or indirectly influence and oversee the management of HCPs were recruited to participate in in-depth interviews through convenience and snowball sampling. Participating health facilities were purposively selected to ensure inclusion of a broad and diverse representation of target population characteristics. Members of the research team had previously established relationships and rapport with staff in each health facility and the research mission of KHRC was known to operational mangers at participating sites. Purposive and snowball sampling techniques were used to recruit representative HCP users of IMCI at these health facilities, including health care assistants, community health officers (CHOs), and community health nurses (CHNs). All HCPs who provided direct treatment to children under five years of age were eligible. Caregivers were recruited using convenience sampling; eligible caregivers included those willing to give consent and attending one of the participating health facilities with children two months to five years of age presenting for an initial visit with an illness that did not require referral to another medical facility and was without symptoms or signs of severe disease (i.e., not able to drink or breastfeed, vomiting everything, lethargy or unconsciousness, or convulsions). Sample sizes were chosen to allow for data saturation in the interview results.

Participating HCPs were brought together in early July 2014 for one day of mPneumonia training, which included the content and functioning of the mPneumonia tool as well as how it should be used in the health facilities. There were also demonstration and practice sessions on how to use the breath counter and pulse oximeter. Of note, since pulse oximetry is not included in the current IMCI algorithm and the participating HCPs had no previous training or experience in obtaining, interpreting, or acting on pulse oximetry readings, a reviewing ethics committee requested that the version of mPneumonia used in this pilot study mask pulse oximetry results. Thus, HCPs’ assessment of the pulse oximetry feature was limited to the process of obtaining a reading and to hypothetical situations about how readings could be used during clinical care. Each HCP had the opportunity to practice with the mPneumonia application and tablet during the course of the training. During training sessions at the first two health facilities, six HCPs were selected for usability testing where a researcher directly observed the HCPs using the mPneumonia application, and asked the HCPs to complete a short questionnaire and system usability ratings to gather feedback about their initial impressions of the perceived usability of the mPneumonia application and associated tablet. The five questions used were identical to those from the prior field testing stage of the mPneumonia prototype.[23]

Tablets with the pulse oximeter hardware connected and software settings already preconfigured were provided to HCPs to use in their practice for approximately one month. HCPs were asked to demonstrate mPneumonia routinely on children of eligible caregivers. mPneumonia was demonstrated only after standard clinical evaluation and care was completed, and no information or clinical care decisions were made based on the findings of the mPneumonia application.

After using mPneumonia for approximately one month, HCPs were asked to participate in a 30 to 60 minute long in-depth interview (IDI) guided by a semi-structured data collection guide in a private setting at the clinic. An interviewer trained in qualitative methods led the interview discussion while additional interview team members took notes on participants’ attitude and body language and on the topics that generated more animated discussion. To understand the HCP experience when using the mPneumonia application, the potential advantages and disadvantages, perceived value and acceptability, usability, capacity for integration into current care practices, and other factors relating to uptake, retrospective probing techniques were used to collect qualitative and quantitative usability metrics from HCPs. In addition, participating caregivers were interviewed at their homes after their children had mPneumonia demonstrated by a HCP. All interviews were conducted by two trained qualitative research staff members using pre-tested interview guides and semi-structured questionnaires. All interviews were audio recorded, transcribed verbatim and translated into English, and daily field notes were taken. Transcripts or audio-recordings were not returned to the participants after the interviews, and participants did not review the study results. The study team conducting interviews included three researchers with graduate degrees in public health, all of whom are co-authors on this paper: CTA, JD, and SN. Two were employees of Kintampo Health Research Centre (KHRC) and one was employed by the sponsor, PATH.

Analysis of the data involved manually sorting and summarizing the data using a mixed methods semi-quantitative and qualitative approach. Quantitative data, including the usability metrics and direct observation findings, were summarized using descriptive statistics in Excel. All qualitative data were cleaned, coded, de-identified, and then analyzed using NVivo software and a codebook employing themes identified *a priori*, including opportunities and barriers for introduction of the mPneumonia application, feasibility of replacing the current paper-based method with the application, and perceived value. The data code tree organized individual codes within each theme. Two researchers trained in qualitative methods at the post-graduate level reviewed all the data for input into the final codebook, a process which included identifying and coding emerging themes and included quality assurance checks.

This research was reviewed and approved by both the PATH and KHRC research ethics committees. This human subject research was conducted according to the principles expressed in the Declaration of Helsinki, and written informed consent was obtained from all study participants.

## Results

A total of 72 people were interviewed as part of this study: 9 district health administrator stakeholders; 30 HCPs; and 30 caregivers. No one enrolled in the study withdrew or dropped out. The HCPs were comprised of CHNs (53%), CHOs (20%), midwives (13%), health assistants (7%) and those with other roles (7%), with a median of 3 years of clinical experience (interquartile range [IQR] 1–5) and a median of 2 years of professional training. HCPs demonstrated mPneumonia with a median of 9 to 10 patients (IQR 2–20). Table 2 describes characteristics of the interviewed HCPs, the facilities in which they worked, and their baseline use of IMCI paper-based protocols. An additional 7 HCPs were involved in usability testing during the training sessions prior to mPneumonia being deployed at the study sites.

**Table 2:** Participant Baseline Characteristics.

Description	N	No. (%)	Median (IQR), minimum-maximum
*Health Care Provider Characteristics*			
Role	30		
Community Health Nurse		16 (53)	
Community Health Officer		6 (20)	
Midwife		4 (13)	
Health Assistant		2 (7)	
Other		2 (7)	
Clinical experience (years)	30		3 (1, 5), 0.25–15
Professional training (years)	30		2 (2, 2), 1.5–2.5
IMCI training completed	30	28 (93)	
*Child Characteristics*			
Age (years)	30		2.5 (1.5, 3), 0.67–4
Health complaint on presentation*	30		
Fever		24 (80)	
Cough		11 (37)	
Vomiting		9 (30)	
Diarrhea		7 (23)	
Not eating well		6 (20)	
Headache		3 (10)	
Stomach ache		2 (7)	
Other		7 (23)	
*Facility Description*			
Facility type	11		
Health center/clinic		6 (55)	
CHPS center		5 (45)	
Electricity available at facility	30	19 (63)	
Electricity outages	21	20 (95)	
Generator available	23	1 (4)	
*IMCI Use at Baseline*			
IMCI protocol used at facility	30	27 (90)	
Time to complete paper IMCI (minutes)	28		12.5 (5, 20), 1.5–40
Routinely skips steps in IMCI	28	18 (64)	

*Multiple responses accepted

### Feasibility of mPneumonia

#### National-level support critical for implementation

Both health administrators and HCPs thought the mPneumonia application would be organizationally and technically feasible to implement. From an administrative perspective, the stakeholders at the district level believed that it would be relatively straightforward to introduce the mPneumonia application in frontline health facilities, as long as its use was approved by the national Ministry of Health decision-makers. A district health officer noted that decision-makers at the national level were “eager” to roll-out information communication technology.

#### Easily integrated into routine childhood care

None of the HCPs in the study had previously used mobile applications for clinical care. After using mPneumonia for one month in clinic settings, all but one interviewed HCPs believed the application could be integrated into the routine clinic care to help with the correct diagnosis and treament of childhood illnesses (Table 3). One HCP summarized this perspective by saying, “[mPneumonia] will help diagnose diseases of the children quickly and it won’t disrupt work. It is very good and does not waste time.” Another noted, “it won’t disturb the flow of the visit.” All HCPs interviewed agreed that providers at their level had adequate training to implement the mPneumonia application. Thirty percent of HCPs noted unprompted that although feasible to introduce, the application would add to their workload as it took more time to complete the application steps than standard practice.

**Table 3:** Health Care Provider Perceptions of mPneumonia.

Description	N	No. (%)	Median (IQR), minimum-maximum
mPneumonia feasible to be integrated in the facility	30	29 (97)	
mPneumonia breath counter makes assessment easier	27	26 (96)	
mPneumonia pulse oximeter makes assessment easier	25	24 (96)	
Time to become comfortable with mPneumonia (days)	23		1 (1, 1), 0–7
Time to complete mPneumonia (minutes)	27		10 (7.5, 22.5), 7–50
mPneumonia faster to use than paper IMCI protocol	20	13 (65)	
Would use mPneumonia if available	30	30 (100)	

#### Electricity needs biggest barrier to use

Health administrators identified the primary barrier to introduction as limited access to electricity in health facilities, a concern with the feasibility of using mPneumonia that interviewed HCPs echoed. “Lack of electricity to charge the device was a problem,” one HCP said. Almost 40% of the sites in the study area did not have electricity. Of the health facilities with electricity available, HCPs reported power outages at 95% of the clinics. When electricity was unavailable at the health facility, HCPs would have to take the devices to the town center, typically a distance of about 15 kilometers on average, in order to charge the batteries. One HCP suggested that extra devices would need to be on hand in the clinic to help cover periods of power failures when the tablets could not be charged, “Because when you are using one [tablet] and the battery runs down, then you can use the others…You can use it till the light comes [back on].” The logistical challenge of charging the tablets also was a deterrent to downloading mPneumonia onto their personal phones to use in the clinic for almost a third of the HCPs interviewed. One HCP expressed this concern by saying, “I have to get the tablet so I will spare the battery of my phone.”

### Usability of mPneumonia

#### Easy-to-use design required little initial training

The general opinion of HCPs was that the mPneumonia application was user-friendly and efficient to use. All HCPs interviewed reported using mPneumonia was a good experience, and that they would use the application again if it was available. The simple design was “not difficult to use… [Y]ou just read and follow the instructions.” Almost 80% of the HCPs said it took them a day or less to be familiar with the device and application when they used it for the first time during the training sessions. Two HCPs said that they only became comfortable with mPneumonia after they had the opportunity to use it with patients, “but after that, it wasn’t difficult again.”

The usability metrics obtained from 7 HCPs at training correspond to the 30 HCPs’ reports of usability after using mPneumonia for one month (Table 4). The group of 7 HCPs for initial usability testing prior to training was split between those with and without prior familiarity with tablets, but the HCPs’ initial perceptions of the application and device did not vary much by prior exposure to tablets. All 7 either “agreed” or “strongly agreed” that the tablet was an appropriate size and shape, and they would use the tablet with pediatric patients if it were available. Six of the 7 HCPs initially thought the application looked easy to use; the one exception was a HCP without prior familiarity with tablet technology who nonetheless noted “if you follow the instructions, it will be alright.” Direct observation of HCPs using the application for the first time identified 5 of the 7 HCPs making critical errors that the participant did not notice themself, including difficulties launching and completing various steps for the application, lack of familiarity with certain IMCI terminology, and data entry errors (Table 5). All 7 HCPs observed using the application for the first time were able to recognize and correct non-critical errors they made, the most common of which was using the pulse oximeter. The orientation of how to open and place the Nonin pulse oximeter probe on a child’s finger was not intuitive to many HCPs as the cord was attached to the same side of the probe as the opening. A pictogram of the child’s finger attached to the pulse oximeter probe was helpful for some, but not all HCPs. Other diagrams in the application were used by HCPs to understand how to use the tablet itself (e.g., identifying low battery) and identify clinical signs (e.g., palmar pallor). Overall, these HCPs thought that with a little practice, mPneumonia would be simple to use and implement during patient care.

**Table 4:** Usability Metrics.

Survey item	Health care providers’ responses									
	A	B	C	D	E	F	G	Mean	Average Response	SD
I am familiar with a tablet. This is a tablet.	1	2	4	2	4	5	2	**2.9**	**Neither agree nor disagree**	1.36
The shape of the tablet is appealing.	5	4	5	4	4	5	4	**4.4**	**Strongly agree**	0.49
The size of the tablet is just right.	4	4	5	4	4	5	4	**4.3**	**Agree**	0.45
If it were available, I would like to use the tablet when I see pediatric patients.	5	5	5	5	4	4	4	**4.6**	**Strongly agree**	0.49
The application looks easy to use.	4	4	4	4	4	5	2	**3.9**	**Agree**	0.83

Note: 1-Strongly disagree; 2-Disagree; 3-Neither agree nor disagree; 4-Agree; 5-Strongly agree

**Table 5:** Task Analysis Results from Direct Observations.

Application Element	Most Frequently Observed Error	HCP Committing Error						
		A	B	C	D	E	F	G
Survey design	Correcting answer selections			NC		C	C	C
	Weight-for-height graph interpretation						NC	
Pulse oximeter	Positioning fingertip probe					NC		
	Launching pulse oximeter	C		NC		NC		
	Recording results	NC				NC		NC
	Connecting pulse oximeter hardware	NC				C		
Breath counter	Launching the breath counter	NC		NC				
IMCI content	Understanding IMCI terminology and subject matter (e.g., stridor, palmar pallor)					C	C	
Software navigation	Launching mPneumonia application				C			
	Minimizing pop-up keyboard		NC		NC			
	Page completion, advancing to next step		NC		NC	C		
Tablet	Turning on tablet	NC						
	Password entry			NC				

Note: HCP-health care provider; NC-noncritical error (noticed by HCP and corrected); C-critical error (unnoticed by HCP)

#### Assisted technology improvement over manual assessments

During the one month pilot study, both the HCPs and the caregivers viewed the pulse oximeter and the breath counter in a positive light, and HCPs found them “easy to use.” All but one of the 30 HCPs who used these tools with patients reported that they made assessments easier. When describing their positive experiences with the pulse oximeter, HCPs mentioned the heart rate count that the pulse oximeter returned was useful and preferred over manual counting. Additionally, HCPs appreciated that the breath counter simplified the process of counting a child’s respiratory rate. One HCP explained the benefit of the counter’s built-in timer saying, “it’s not like the manual [method] where we used to do the breath counting that you have to look at the client and at the same time look at your wrist watch. So, you begin the two things at the same time.” The majority of HCPs believed that these tools will help better detect illness and “reduce inaccuracy.” The main challenge identified in using the pulse oximeter was that it required the child to be still because “if the child is not calm, it will be difficult to read” as the child’s movement frequently prevented displaying the results. Movement of the child also was a challenge for using the breath counter. One HCP explained that children “struggling” is an inherent problem with counting respiratory rate by saying, “with the children, [counting breaths] is not easy, but compared to what we usually do, the device is better…There is nothing you can do about the machine—it is the children.” Four HCPs thought that the breath counter could be improved if it was automatic and would generate a respiratory rate without any human counting. One explained, “I think [the breath counter] will be easier when the meter is attached to the child, so that it will take the reading for you.”

#### Time constraints with full assessment

When asked generally about barriers to the usability of mPneumonia, the primary barrier identified by HCPs was the time it took to complete the application. The direct observation of 6 HCPs during the usability testing portion saw a median of 25 minutes (mean of 28 minutes) to complete the application for the first time, with a range of 20 to 50 minutes. When HCPs were asked about completion times after using mPneumonia for one month, the median reported time to use mPneumonia with a patient was 10 minutes. Four HCPs reported that it took 30 minutes or more to complete mPneumonia, especially if the caregivers’ responses to the application questions were inadequate and needed further probing by the HCP or if the application itself crashed, was slow to load, or did not save the information properly. One of the HCPs who took 30 minutes to complete the application explained that the application “is very delicate, so they should make it stronger than it is because it easily goes down.” Many HCPs mentioned the fact that the time it takes to go through the application steps may prevent caregivers from allowing the application to be used on their children. “One of the barriers is that if the staff are not many and the mothers are in a hurry to go home and do other things. I wonder whether they will be willing to let us use the application on their children.” Another HCP summarized the reality of using the application in a busy clinic by noting, “there may be several patients outside waiting and you will be in the room for about 30 minutes with one patient when using the device before you prescribe drugs. That will be a challenge.” Despite this perceived barrier, 90% of HCPs preferred the mPneumonia application to paper-based IMCI protocols.

Although the time to complete mPneumonia repeatedly surfaced during the course of the interviews, when asked directly to compare mPneumonia to paper-based IMCI protocols, 65% of respondents said that mPneumonia took less time. Of 20 HCPs asked, 13 said mPneumonia was faster and 7 said it took the same amount of time. The median reported time to use paper-based IMCI protocols with a patient was 12.5 minutes. HCPs that noted the application took longer to complete than the paper-based IMCI protocols also commented that it was more complete—not allowing one to skip any steps—and thus was the “safest” and “best option” for diagnosing a child. Ninety-three percent of the HCPs had completed training in IMCI, but 64% of the HCPs interviewed reported skipping steps while assessing patients using paper-based IMCI protocols. One HCP summarized the common finding that it was easy to learn from the application itself while assessing children by saying, “It was a good experience because it helped me to know more and at the end it teaches you how to counsel the mother on nutrition.”

### Acceptability

#### Trusted technology by providers and caregivers

All but one HCP interviewed thought the application was accurate, and all 30 HCPs thought it was appropriate for diagnosing childhood pneumonia. The mPneumonia application was acceptable to both HCPs and caregivers due to the users’ desire for “new” and “modern” tools, trust in the accuracy of the technology, and appreciation for the application’s thoroughness as compared to the standard of care. In particular, caregivers liked the non-invasive aspect of the application. Challenges for the acceptability of mPneumonia revolved around confusion on the part of caregivers about the application and its introduction.

HCPs spoke about the novelty of using the devices as a positive aspect of the application for caregivers’ acceptance of its use with their children. Being new was associated with the perception of improved care. An HCP noted that “when you use it on the child, the mother becomes excited. She sees that we are doing something new on the child and for her it means the facility is improving.” Interviewed caregivers noted similar thoughts on perceiving the newness of the application as a draw. The majority of caregivers were pleased with the application and saw its use as a sign of modernity and current trends in medicine: “right now, the way the world has progressed, it is this modern machines that everyone is using.” HCPs believed that the use of the devices would help generate demand for health care services due to increased care-seeking behavior. Multiple caregivers emphasized spreading the word about this new tool at the health clinic so that others could also benefit. A representative quote from a caregiver stated, “I thought the new machine will help to diagnose illnesses and so I told my friends that there is a new device at the clinic so they should send their children to the facility when they are not well.”

#### Automated algorithm perceived as accurate

HCPs believed that the mPneumonia application would help them make the correct diagnosis and provide the right treatment to the children. They trusted the results of the mPneumonia algorithm, reporting that “the machine does not tell lies, it will rather tell you the right thing to do.” HCPs also valued the results from the breath counter noting, “If you use the manual counting of the breath, you might make a mistake but this one is a machine so you get the accurate counting.”

Similarly, caregivers expressed a great deal of confidence in technology in general, which extended to the mPneumonia application itself. Multiple caregivers believed that a result from a “machine” was superior to assessments done by HCPs unassisted. One caregiver expressed this by saying, “I know that the possibility of the machine making a mistake is small. Even though machines were made by human beings, they are more accurate than human beings.” Some caregivers appeared willing to accept the new mPneumonia application because they did not have faith in the care that children would otherwise receive.

Many caregivers liked the way the mPneumonia application systematically “checked” their child’s symptoms, noting that it could potentially uncover a problem or sign that may have been missed with the usual care routine. In particular, caregivers linked the systematic review of their child through the application’s algorithm with improved treatment outcomes. Most of the caregivers believed that the application would help detect the cause of their child’s illness, enabling the child to get the correct treatment. One said, “if the device is used on the child, it will be able to detect the kind of illness and give appropriate treatment. At times, if you visit the health facility they are not able to know the illness of the child so as to give the right treatment, but the device may find out the illness.”

#### Non-invasive aspect important to caregivers

HCPs were sensitive to caregivers’ desires not to have invasive techniques such as blood draws, so the fact that mPneumonia is entirely non-invasive was an attractive quality of the application. One HCP explained that the pulse oximeter would be acceptable to caregivers by saying, “They [caregivers] will like it because it will not involve any blood draw.” A fear of blood samples was so pervasive that some caregivers were initially scared of the application because they thought the device would be used to take a blood sample from their children. This fear was observed in the children as some were “crying and then resisting” when the HCP would attach the pulse oximeter.

#### Education importance for acceptance

In addition to training for HCPs on how to use the device, HCPs thought that caregivers also needed information and counseling on the new application’s purpose and procedures. The newness of the device had been a positive for most caregivers, while a negative for a minority. The women who brought their children to the clinics had not seen the mPneumonia application before and this made some of them “very confused” as to why the device was being demonstrated with their children and caused them to wonder what exactly the application would do for their children. One expressed the concern that they thought their children’s condition was *“*serious to the extent that they had to use a machine” to diagnose their illness. A few caregivers felt using the application made the children uncomfortable, in particular when the child needed to stay still for the pulse oximetry and breath count assessments.

## Discussion

Throughout the world in LRS, present-day standards of care for pneumonia and other childhood illnesses rely on the correct use of WHO IMCI and iCCM protocols. Yet, application of WHO algorithms is imperfect due to a lack of trained HCPs, supervision, and financial resources.[3, 5–7] In practice, HCPs frequently do not adhere to the WHO guidelines, and clinical assessments are incomplete and inaccurate.[6, 25] mPneumonia is constructed to minimize the need for training, enhance adherence to the IMCI algorithm, and offer clear guidance for interpreting results. The step-by-step software improves adherence to the algorithm by not letting HCPs skip essential assessment questions, allowing lesser-trained HCPs to diagnose children with pneumonia and other childhood illnesses, and those at highest risk of death and in need of immediate treatment. The mPneumonia breath counter permits the HCP to focus exclusively on observing and recording each breath without also needing to keep track of the count or the time. mPneumonia utilizes a pulse oximeter to identify hypoxemia, allowing more effective and cost-effective use of oxygen, a lifesaving therapy.[26] Pulse oximetry can be valuable even in the absence of supplemental oxygen because it can be used to prioritize or urgently refer patients to facilities with more intensive care.[27]

The results of the pilot study in Ghana provided valuable information for understanding the feasibility, usability and acceptability of mPneumonia. Strong interest in technology suggests district- and community-level support for mobile application use. The district health administrators’ perspectives highlighted the need to actively engage national health systems and follow national policies and decision-making when introducing new technology into care. The pilot study showed that in addition to being organizationally and technically feasible to implement and integrate into routine clinical care, mPneumonia was thought to be user-friendly and efficient, to improve accurate diagnosis and treatment, and to increase care-seeking behavior. These benefits were seen among HCPs with an average of 2 years of professionl training. Enhancing HCPs’ knowledge of pneumonia diagnosis and treatment made using the application a rewarding experience, and HCPs reported that they would use the application again if it was available. Gaps in HCPs’ familiarity with IMCI were identified during direct observation of HCPs using mPneumonia and in interviews after one month of use, suggesting HCPs could benefit from pairing mPneumonia use with refresher IMCI trainings. Additionally, the pictures embedded in the application were shown to be helpful for trainining HCPs during the initial usability testing, suggesting that further graphics and/or embedded videos of clinical signs could also provide an expanded benefit for mPneumonia by way of enhancing provider training. Both HCPs and caregivers expressed increased trust and confidence in the application over standard care. It is unclear from this study’s findings how much of this trust was due to the performance of mPneumonia iteself versus a seemingly pervasive trust in new technology as representative of modern medicine advances.

Reported potential barriers included electricity requirements for charging and the time needed to complete the application. Even in settings with limited and/or intermittent electricty, HCPs found enough value in the mPneumonia application to identify solutions to meet the tablets’ charging requirements, even if it meant bringing the equipment into town after working hours. HCPs’ perception of the time to complete mPneumonia was identified as a barrier, even though the median time to complete mPneumonia after one month of use was reported as shorter than the median time for paper-based IMCI protocols. Notably, in this pilot study, mPneumonia was only demonstrated with patients after they had received clinical assessments per the standard of care in the clinic, so using mPneumonia did add time to a patient’s visit, even if using the application itself was comparable to the time of paper-based protocols. This additional evaluation and the time necessary to complete it may have influenced the HCPs‘ and caregivers‘ perceptions of how much time was necessary to use the mPneumonia tool. Additionally, the inconsistency in perception of completion time could be explained by the change over time of how long it took; the median time to complete mPneumonia dropped from 25 minutes observed during the initial usability testing to 10 minutes reported by HCPs after one month of use. The feature of mPneumonia that would not allow a user to skip steps of the IMCI algorithm also meant that a HCP could not adapt an assessment of any child to accommodate especially high patient loads during busy periods at the facility. Despite this, it is noteworthy that 90% of HCPs preferred the mPneumonia application to paper-based IMCI protocols. Updates to the mPneumonia application from the initial prototype reduced the time it took HCPs to complete the application the first time, cutting it by over 40% from a mean of 43.4 minutes (previously reported) to a median of 25 minutes (reported here).[23] Additional improvements to the application were also made based on the pilot test results.

Another limitation to this pilot study was that we were not testing the pulse oximeter among health care providers familiar with pulse oximetry, and so the full use and benefit of pulse oximetry were not apparent to the users. As pulse oximetry is not included in the current IMCI algorithm and was not previously a part of the participating HCPs’ training or standard care practice, it was beyond the scope of the study to validate any HCP training in the interpretation of pulse oximetry results, and thus, the pilot study masked the pulse oximetry readings. One consequence of this unfamiliarity with pulse oximetry is that the HCPs only considered possible applications for the heart rate counter aspect of the pulse oximeter and HCPs did not provide meaningful input on how pulse oximetry readings could potentially enhance or guide care at their health center. Moving forward it would be important to use and test unmasked pulse oximeters and provide adequate training in their use. More importantly, because pulse oximetry is critical in the management of childhood illnesses, it should be included in appropriate policies, guidelines, trainings and practice.

## Conclusion

Introducing the mPneumonia application in frontline health facilities in Ghana was feasible to integrate within current HCP practices and was found to be both usable and acceptable among HCPs and caregivers for diagnosis and treatment of young children two months to five years of age. A potentially promising new tool for improved health care delivery that is designed to be a free, open-source platform easily adapted for various countries and/or contexts, mPneumonia integrates a user-friendly step-by-step guide to increase adherence to WHO diagnostic protocols, incorporates objective measurements such as respiratory rate and pulse oximetry, and offers decision-making support. The software can integrate with other ODK-based tools and does not require an internet connection to operate. The integrated pulse oximeter is FDA-approved for medical grade use and is competitively priced. As new childhood pneumonia innovations are developed and adapted, it is critical to prioritize the needs of HCPs and caregivers, and understand the constraints of using this technology in a LRS. mPneumonia has the potential to facilitate prompt diagnosis and assessment, decrease the risk of death due to delayed treatment, and have an impact on the leading causes of childhood mortality. Future expansion of mHealth technology for IMCI can build upon the lessons learned in this evaluation.

## Supporting Information

S1 Manual mPneumonia user manual. (DOCX)

S1 Table COREQ checklist. (DOCX)

S2 Table Health care provider dataset. (XLSX)

S3 Table Caregiver dataset. (XLSX)
