# Supplementary material for: mPneumonia, an Innovation for Diagnosing and Treating Childhood Pneumonia in Low-Resource Settings: A Feasibility, Usability and Acceptability Study in Ghana
Source: PLoS One. 2016 Oct 27;11(10):e0165201. doi: 10.1371/journal.pone.0165201 (PMC5082847; doi:10.1371/journal.pone.0165201)

S1 Manual. mPneumonia user manual

mPneumonia Application Operating Manual

# FOR RESEARCH USE ONLY

WARNING:

- *The mPneumonia application is for research use ONLY.*
- *The mPneumonia application should NOT be used for making clinical care decisions.*
- *The mPneumonia application should NOT be used on critically ill patients nor should it delay appropriate clinical evaluation or care. Each child should also be evaluated using standard diagnostic procedures and tools.*
- *Any child with Danger Signs should be EXCLUDED from the use of the mPneumonia application.*
  - *Danger signs: child is unable to drink or breastfeed, vomiting everything, has convulsions, or is lethargic or unconscious.*
- *The mPneumonia application only supports the IMCI protocol for children over 2 months and under 5 years of age.*
- *Only appropriately trained users should use the mPneumonia application.*
- *Users should consult the operating manuals of the various hardware components for the proper use and safety precautions for the hardware components. This manual is NOT meant to provide complete instructions, as it only provides a general overview of how to use the mPneumonia application. Consult product manuals for proper safety precautions.*

## Introduction

The mPneumonia application integrates an easy-to-use respiratory rate counter and pulse oximeter into a user-friendly digital IMCI process in an effort to improve diagnostic accuracy and IMCI protocol adherence. The mPneumonia application is designed to work on an Android device (running version 4.1 or greater) and is composed of several hardware components and software components. To connect the pulse oximeter to the Android device, the Android device needs to have USB master capabilities to communicate with the pulse oximeter.


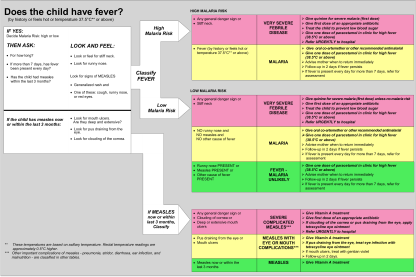

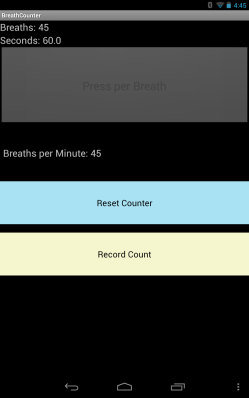

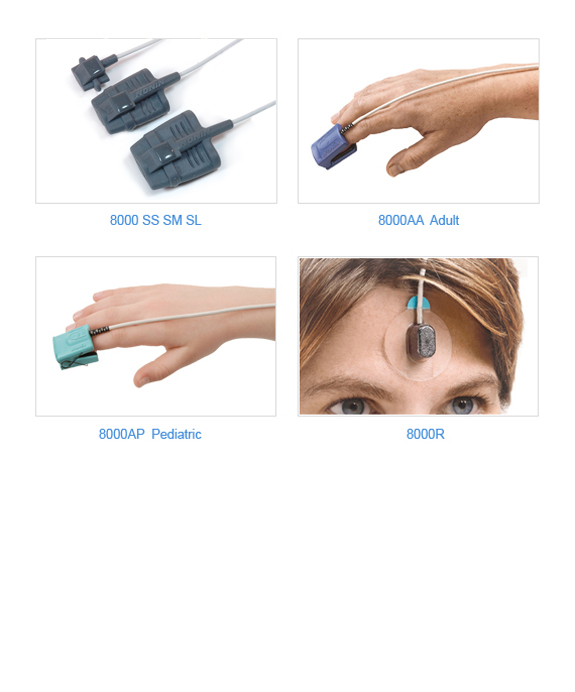


***IMCI algorithm***

***Respiratory Rate Counter***

***Pulse Oximeter***

***+***

***+***

The mPneumonia application leverages the Open Data Kit (ODK) toolkit to implement the IMCI protocol and interface the Android device with external sensors. ODK is an extensible, open-source suite of tools aimed at building information services for low-resource settings. The mPneumonia application is an IMCI-based assessment tool that was created using ODK Survey to implement a patient intake process for diagnosis of childhood illness and digital recording of patient data. ODK Survey renders the application logic as prompts to users to collect data and support rich data input types (e.g., photo, GPS, barcodes). Additionally, ODK Survey provides facilities to customize for localization and languages as well as require questions be answered and control the order of questions, thereby increasing protocol adherence. The digital version of the IMCI protocol in ODK Survey is enhanced by two additional Android applications to: 1) assess respiratory rate, and 2) record the child’s oxygen saturation. The respiratory rate counter is a simple Android application that during a 60 second interval counts the number of times a user presses a button. To use the respiratory rate counter, a user presses the button for each of the child’s breaths; thereby enabling the user to stay focused on the child. The counting application is designed to vibrate to give quiet feedback to the user that their counting of the breath was successful. ODK Sensors’ Framework handles the interface between the USFDA-approved pulse oximeter sensor and the mobile phone.

### Hardware Components of mPnemonia

1. Samsung Galaxy Note II (or other suitable Android device running an OS version 4.1 or higher with USB master hardware capabilities)
2. Nonin pulse oximeter probe setup (specifically Nonin 8000AP Pediatric connected to a Nonin Xpod)

### Software Components

1. ODK Survey Android App (renders the IMCI questions and protocol)
2. ODK Sensors Android App (enables Android to connect with external sensors)
3. PulseOxDriver Android App (enables ODK sensors to interface with pulse oximeter)
4. BreathCounter Android App (is the respiratory rate counter)

## mPneumonia Setup

This section explains how to setup the mPneumonia application. This setup should only need to be performed once to prepare the application. The mPneumonia application is composed of several hardware components and software components.

#### Prepare hardware components

1. Turn on the Samsung Galaxy Note II and make sure the Galaxy Note II’s battery is charged. NOTE: The Samsung Note II must have an SD card installed so that the applications have space to store data.
2. Connect the pulse oximeter finger clip to the Nonin Xpod (as shown in the last figure below circled in red).

| 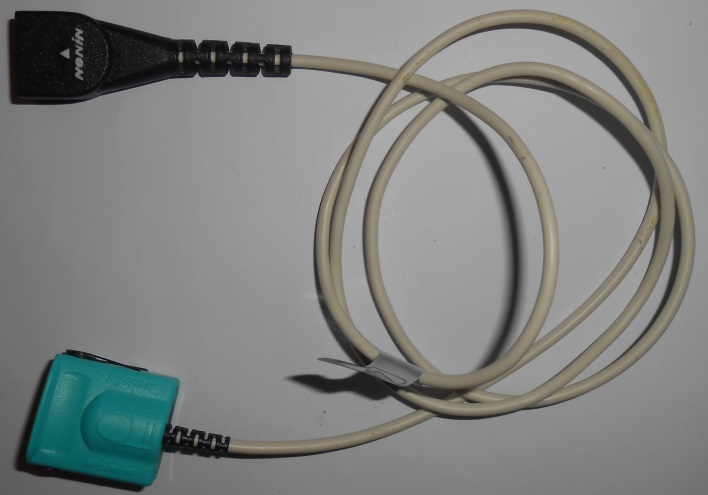  Pulse oximeter finger clip (Nonin 8000AP Pediatric) | 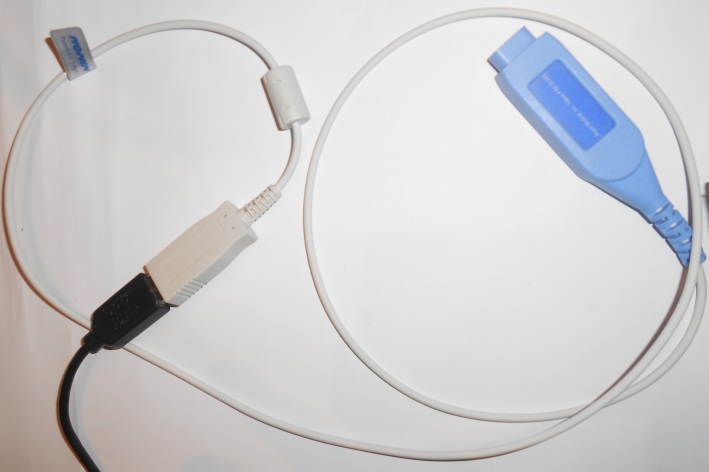  Nonin Xpod |
| --- | --- |
| 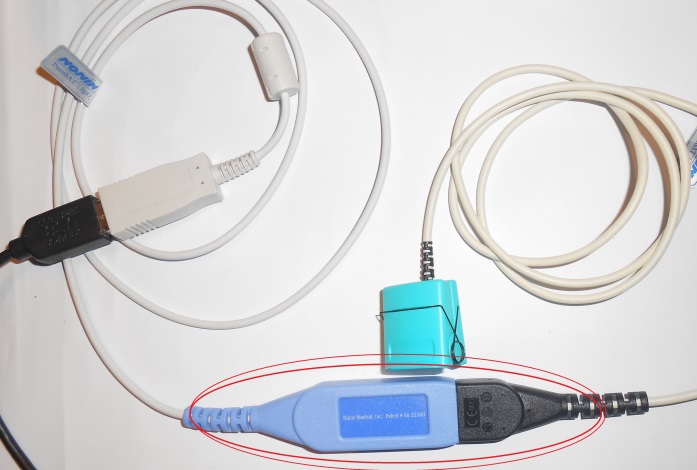  How to connect finger clip to Xpod | |

#### Prepare software components

Note: An APK is the 3 letter file extension used to denote that the file is an Android Application Package (APK) and that the file (denoted by apk) can be installed on to an Android Device.

1. Install the ODK Survey application apk onto the Android phone (download the application from a website or from an application store such as Google Play)
2. Install the ODK Sensors application apk on to the Android to phone (download the application from a website or from an app store such as Google Play)
3. Install the PulseOxDriver application apk on to the Android phone (download the application from a website or from an application store such as Google Play)
4. Install the BreathCounter application apk on to the Android phone (download the application from a website or from an application store such as Google Play)

#### Register pulse oximeter with ODK Sensors Framework and Android

1. Connect the pulse oximeter to the Android phone’s USB port.


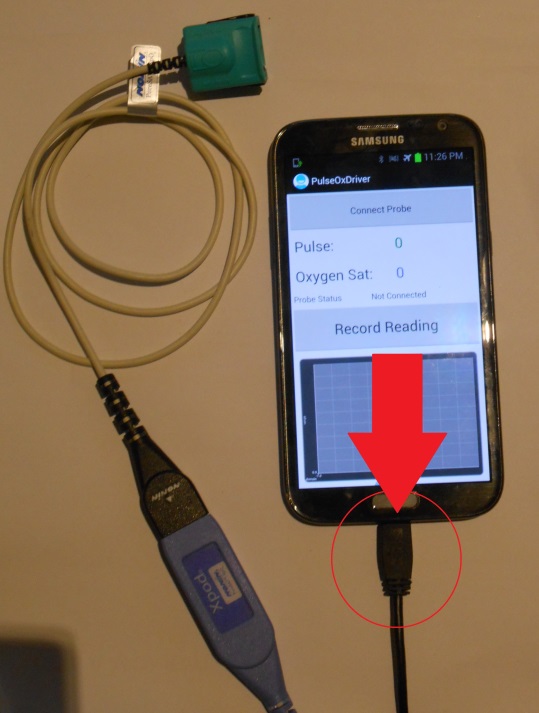


1. Launch the PulseOxDriver application by clicking the icon (circled in red in the left figure below). If the pulse-oximeter probe was properly connected a pop-up will appear from ODK Sensors asking permission to access the USB device. Click ‘OK’ on the pop-up to authorize ODK Sensors to access the pulse-oximeter (this is a requirement of the Android operating system).


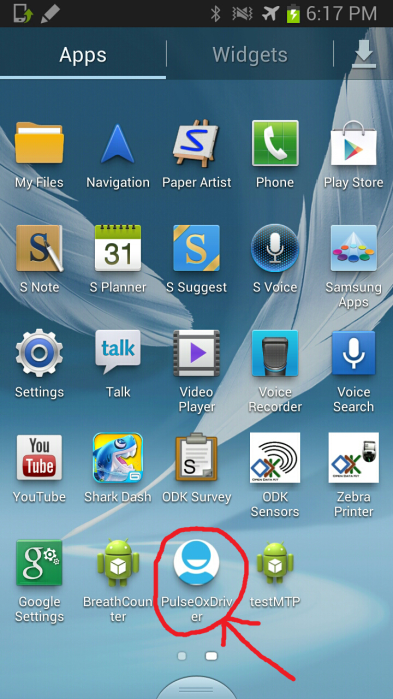

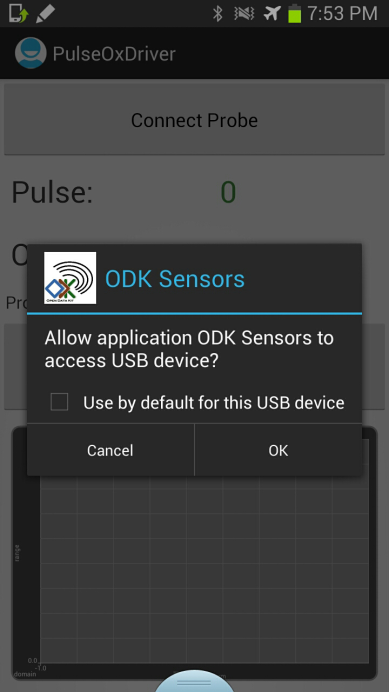


1. Next click the ‘Connect Probe’ button (circled in red in the figure below).


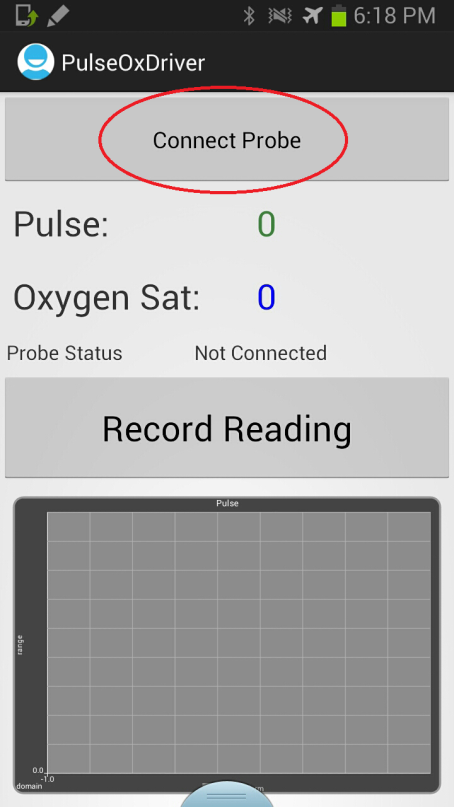


1. The first time a user clicks on the ‘Connect Probe’ button, a pop-up a screen will appear that has two buttons ‘Add USB Device’ and ‘Add Bluetooth Device’ (shown in figure below). This occurs because ODK Sensor framework does not know about the pulse-oximeter yet.
2. To register the pulse oximeter with ODK Sensors Framework, click the ‘Add USB Device’ button (shown with red circle in left figure below). A list of available sensors will appear and in the list should be ‘FTDI’.


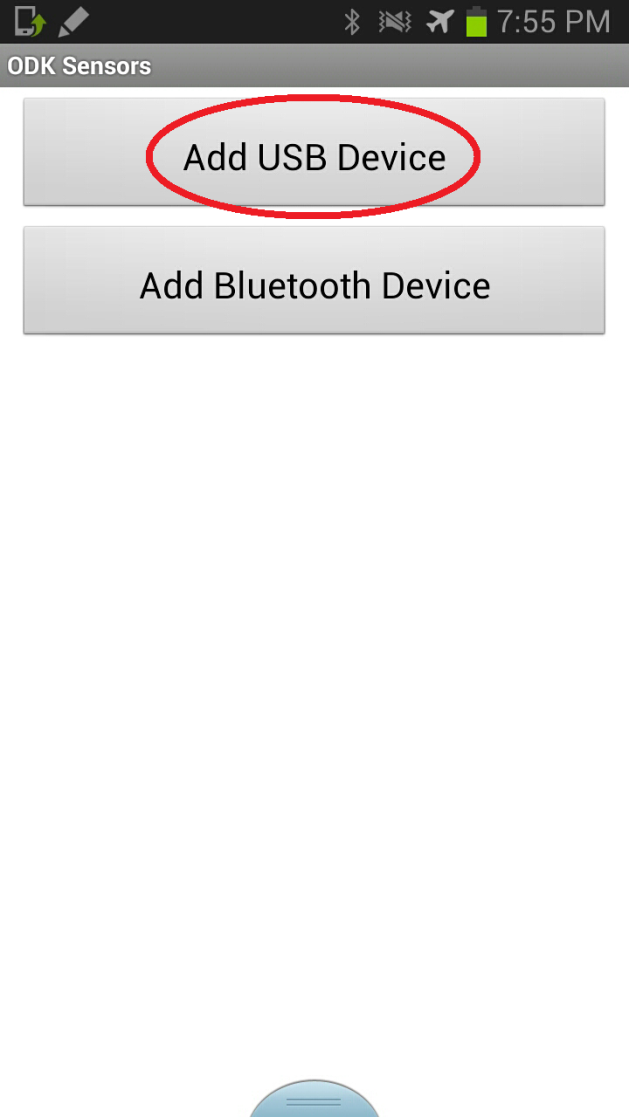

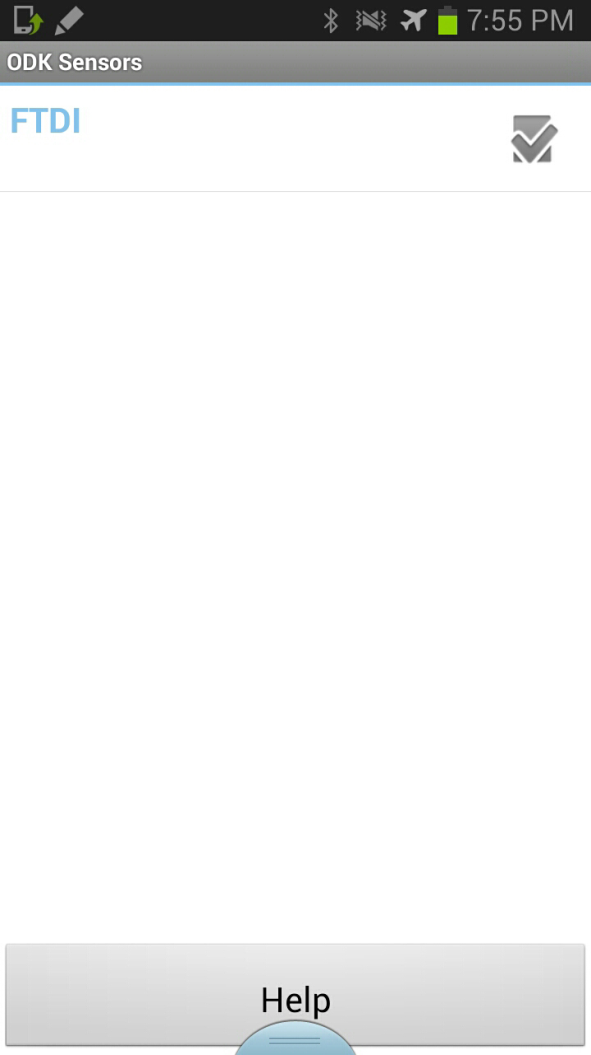


1. Next Click on “FTDI” (shown in red in left figure below) and a pop-up should appear (right figure below).


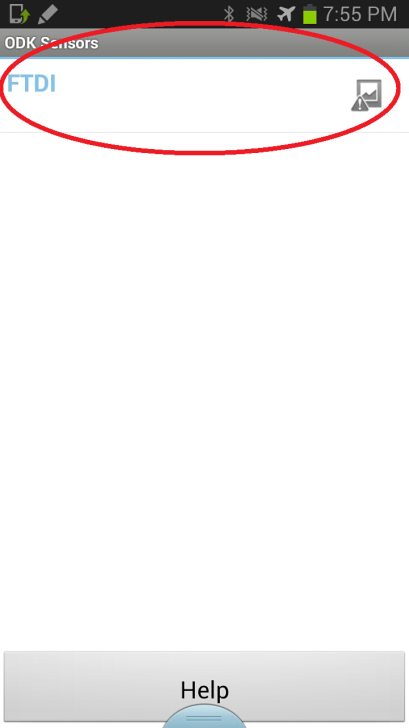

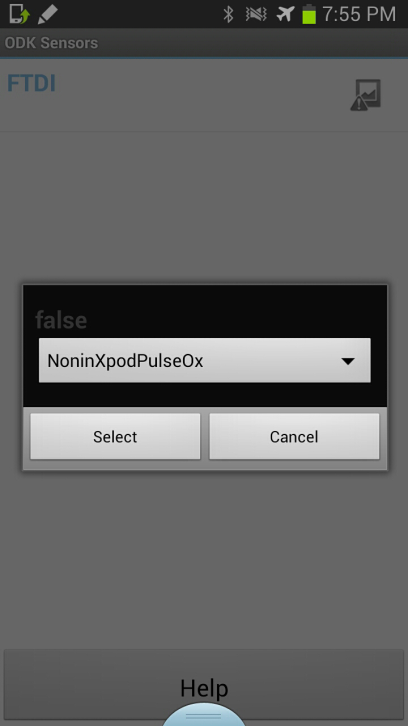


1. Next click/tap ‘Select’ with the following text in dropdown “NoninXpodPulseOx” (should come up as default).
2. Once the pop-up disappears the icon on the right hand-side of the screen should update to show the pulse oximeter is setup (see icons below). Next Click on “FTDI” a second time. This completes the setup process for the pulse oximeter driver.


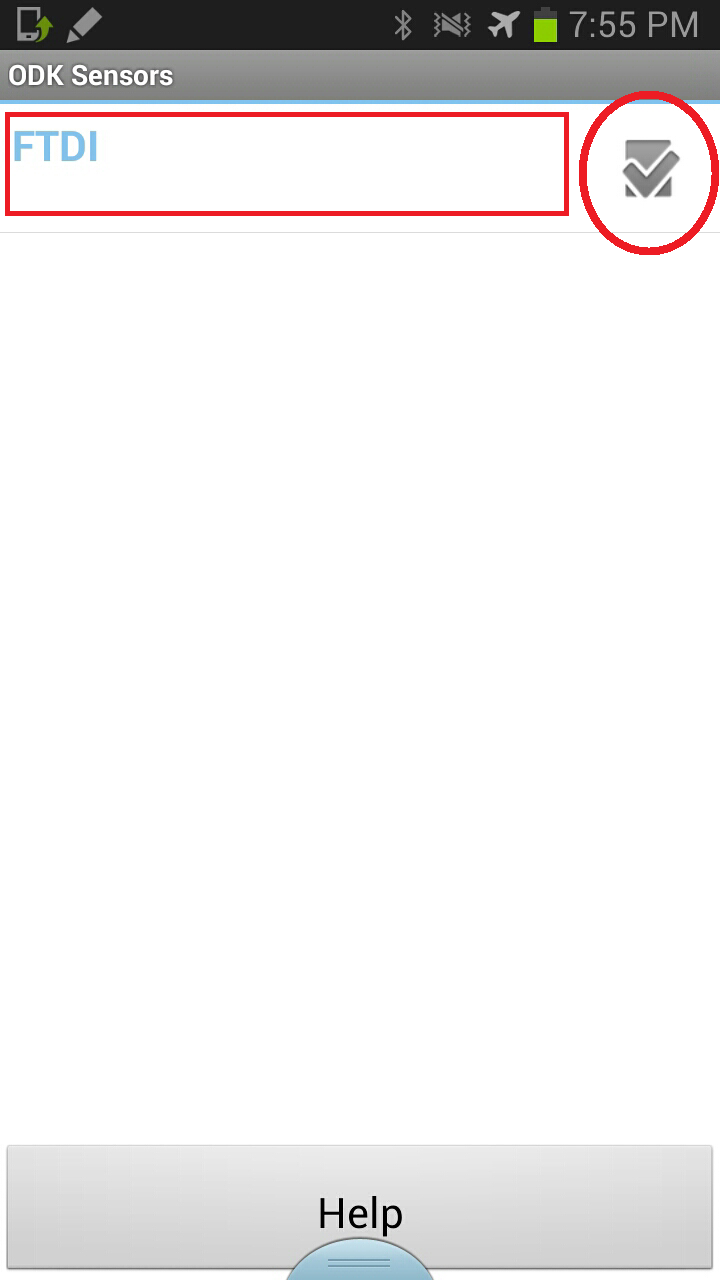


## Launching the mPneumonia application in ODK Survey

To access the mPneumonia application a user needs to launch ODK Survey.

1. To launch ODK Survey click/tap on ODK Survey’s program icon (shown in figure below).


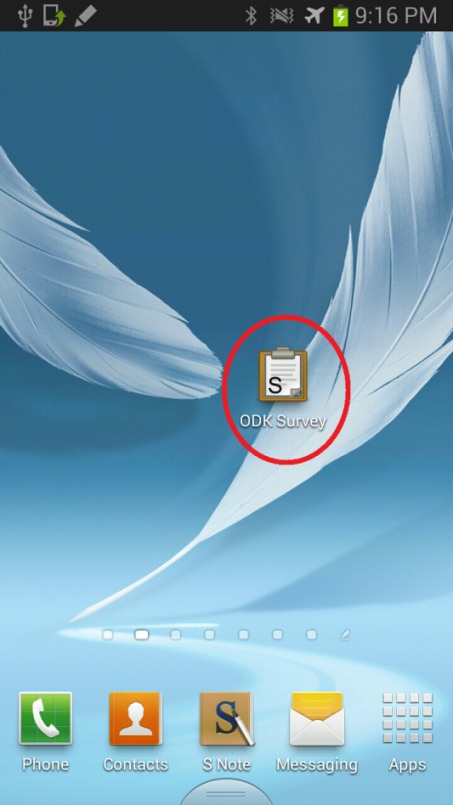


1. Once ODK Survey is launched/opened users will be presented with a list of forms/surveys to fill out. Select the form/survey titled “mPneumonia” to open up theIMCI protocol and begin filling in the question prompts.


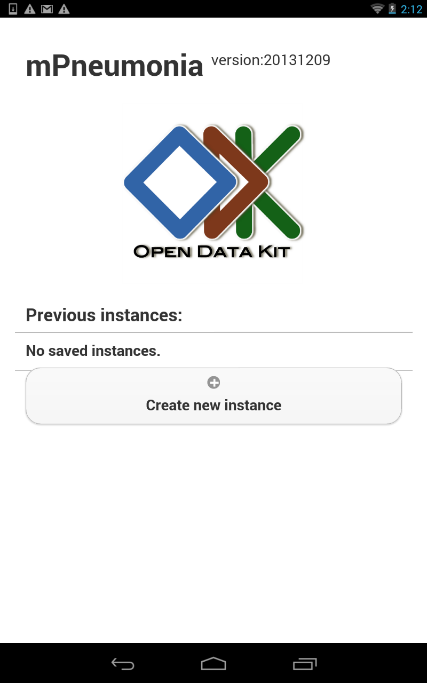

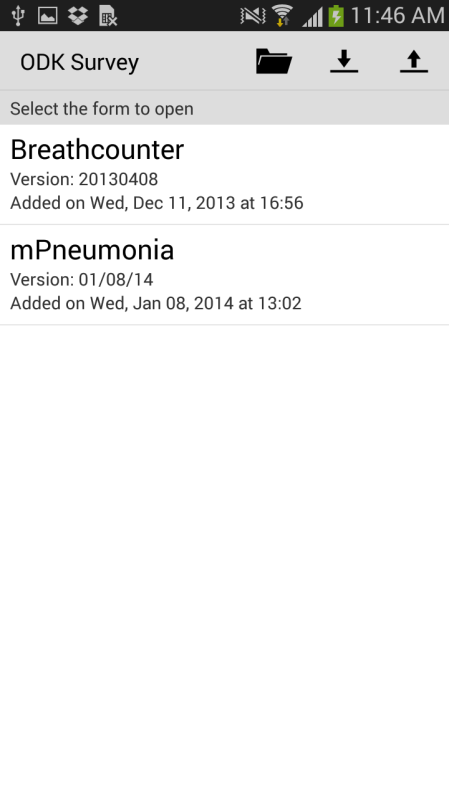


1. To fill in the question prompts simply click on the field to enter and a keyboard will pop up on the bottom of the screen allowing users to enter data. Multiple choice questions can be answered by touching on the choice icon, the choice will be denoted by a color change (‘Not present’ selection is shown in the figure on the right below).


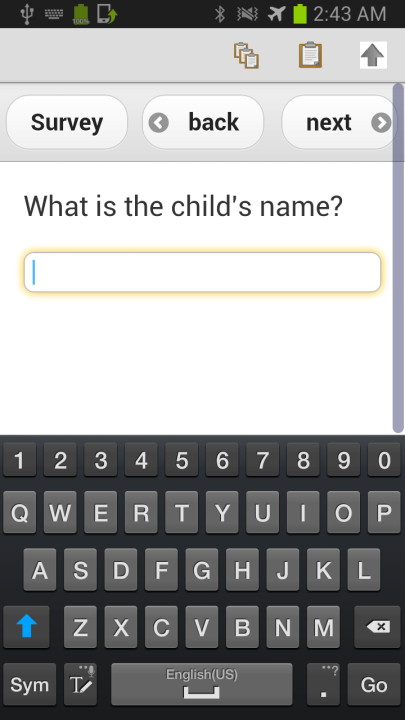

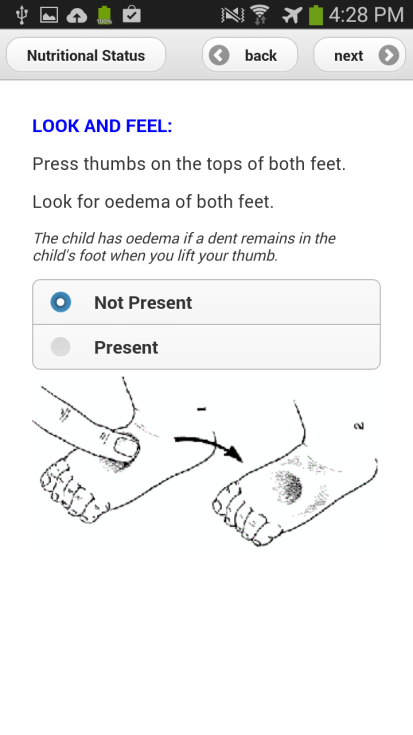


1. Once a user filled out all the questions on the screen the user has two choices to go move on to a different screen with different questions. It’s up to the user to choose their preferred method for question advancement: Option #1: use the back and next buttons located in the bar at the top right of the screen; Option #2: swipe left or right by dragging a finger across the screen either left to right, or right to left depending on the direction the user wants to proceed (dragging the finger across the screen is also known as swiping). If a user tries to advance and a question that has been specified as ‘required’ (meaning required to be answered) by the form/survey designer, the user will receive a pop-up screen(shown in figure below) informing the user the question must answer the questions before proceeding.


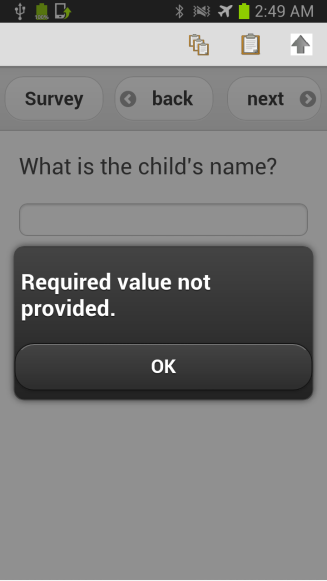


1. As the user advances through the questions ODK Survey automatically determines the relevant questions to show based on the specification of the form/survey designer. Once the required information has been entered the survey will show the classifications as well as give treatment instructions. The navigation of classification and treatment section is the same as the question section.
2. ODK Survey also has a menu that allows the user to exit the survey from the current screen without saving changes or the user can chose to save the changes to come back later to complete the IMCI protocol. To access the menu the user should click/tap the button located near the top left of the screen (shown in red in the figure below on the left). Once this button is clicked/tapped a pop up will appear (shown in the figure below on the right) allowing the user to perform special functions such as exiting or entering the “Contents” menu to navigate between sections of the IMCI protocol.


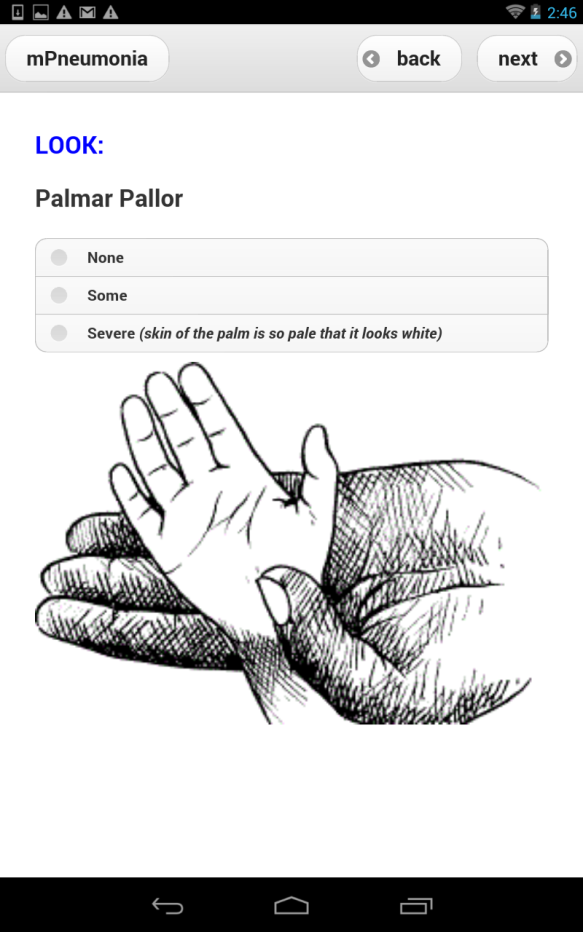

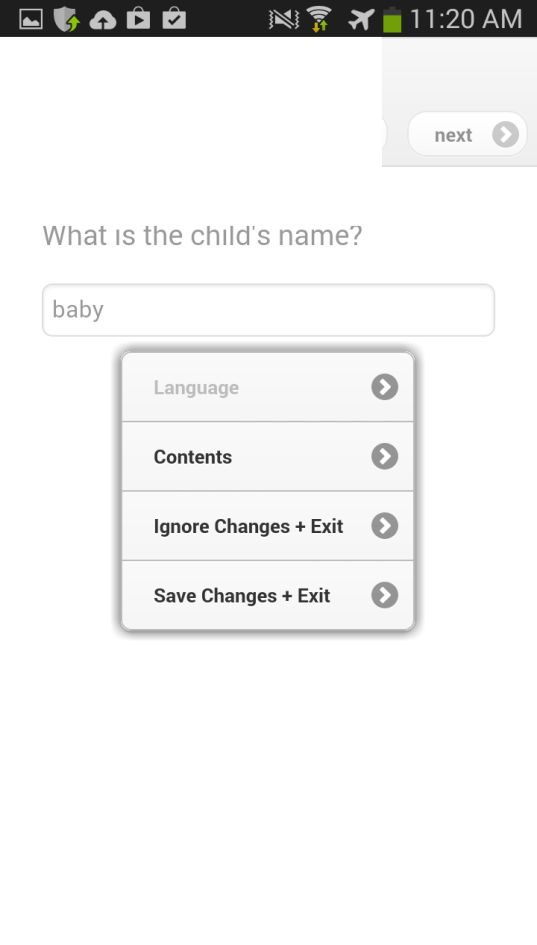


## Obtaining a Respiratory Rate Count

The respiratory rate counter is a simple Android application that during a 60 second interval counts the number of times a user presses a button. To use the respiratory rate counter, a user should press the button for each of a child’s breaths. The 60 second timer starts the first time the button is pressed/ tapped to count the first breath. To help the user stay focused on the child, the counting application is designed to vibrate to give quiet feedback to the user that their tap to count the breath was registered so the user does not need to take their eyes off the child. When the timer reaches 60 seconds the respiratory counter beeps and vibrates for a longer period of time to alert the user the 60 second counting interval is over.

When reaching the “Count the breaths in one minute” question of the mPneumonia application (shown in figure below) perform the following steps.


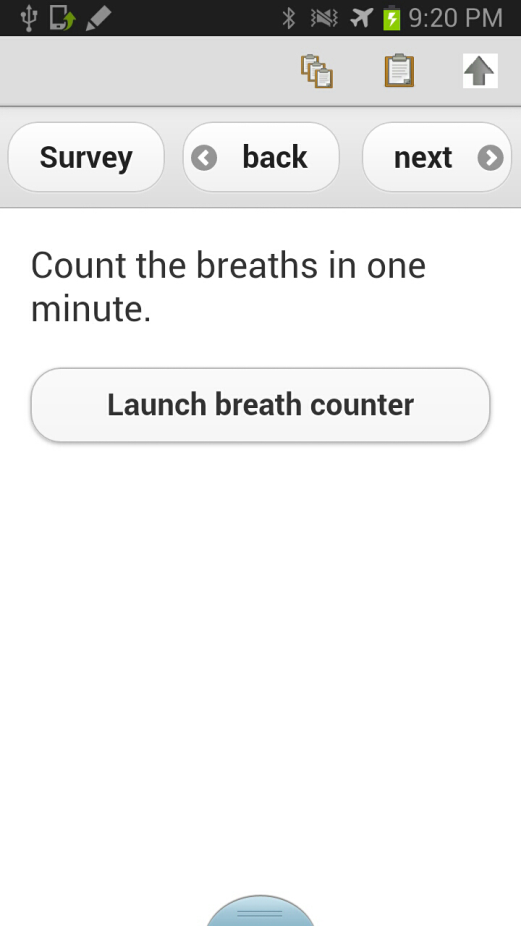


1. Tap the “Launch breath counter” button (circled in red in the below figure on the left). After tapping the button the respiratory rate counter (also known as breath counter) will be displayed (shown in the right figure below).


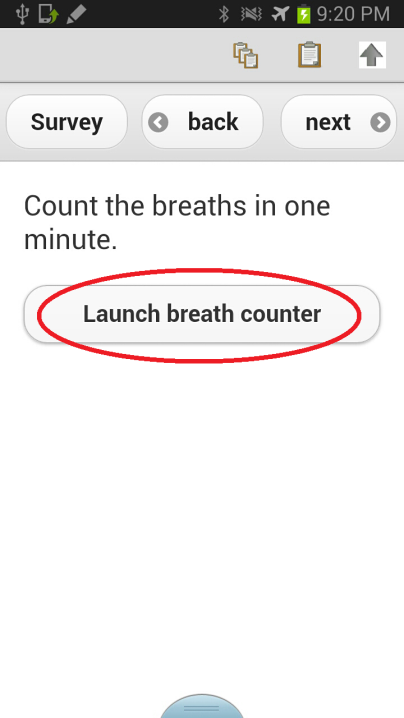

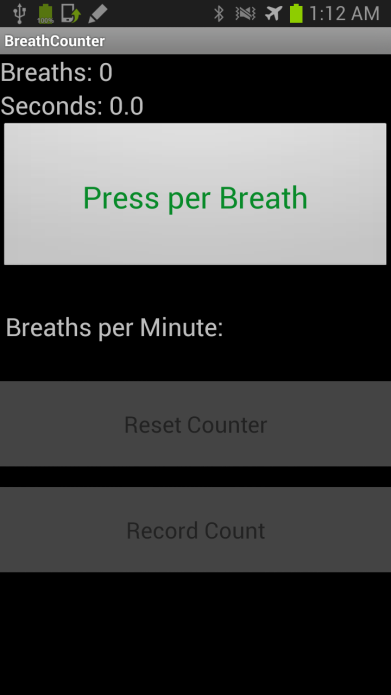


1. To start counting a child’s respiratory rate a user should tap the ‘Press per Breath’ button (circled in red in the figure below). The 60 second timer starts when the first button occurs, this first tap/click should correspond to the child’s first breath that the user wishes to count. To help the user stay focused on the child, the counting application is designed to vibrate to give quiet feedback to the user that their counting of the breath was successful so the user does not need to take their eyes off the child.


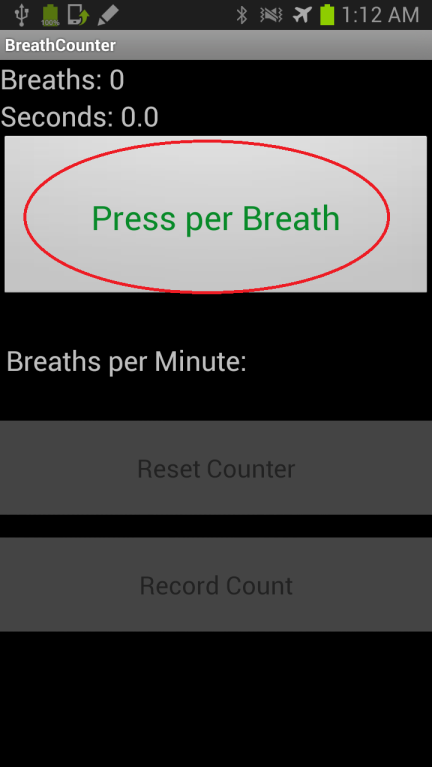


1. The user should press the ‘Press per Breath’ button for EACH of the child’s breaths during the 60 seconds. If a mistake occurs the user can press the ‘Reset Counter’ button and go back to step 2 to restart the counting procedure. At the top of the screen the respiratory rate application displays the number of breaths counted so far as well as the number of seconds elapsed. To help the user stay focused on the child, the counting application vibrates to give quiet feedback to the user that their counting of the breath was successful so the user does not need to take their eyes off the child.


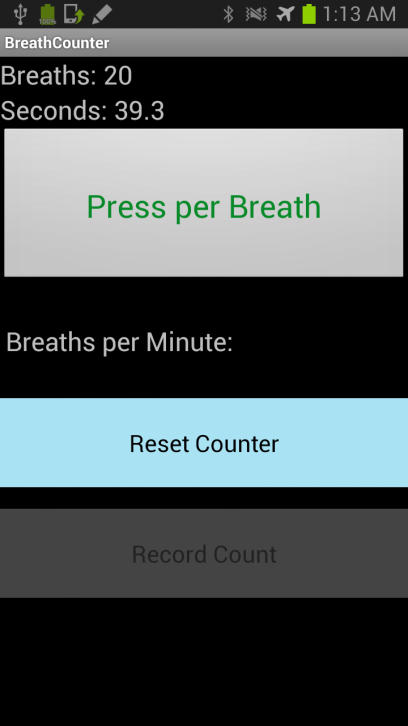


1. When the timer reaches 60 the respiratory counter beeps and vibrates for a longer period of time to alert the user the 60 second counting interval is over. The user then has the option to press the ‘Reset Counter’ button to start again if a mistake was made or press the ‘Record Count’ button to record the respiratory rate value into the IMCI protocol. NOTE: The ‘Record Count’ button is not available until the 60 seconds of counting is complete.


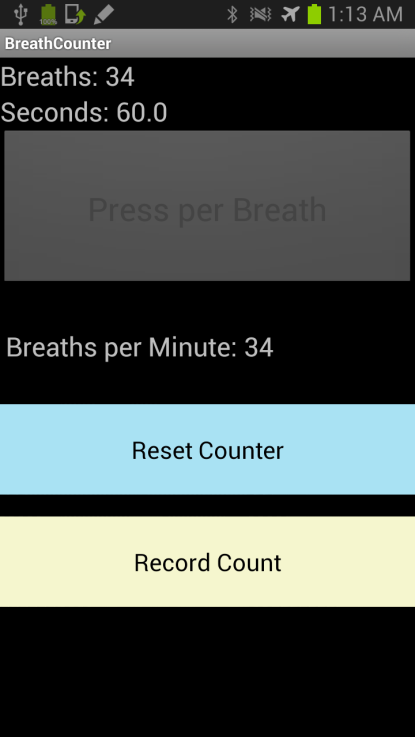


## Obtaining a Pulse Oximeter reading

The Nonin Xpod tool connects to an Android phone using custom drivers that enable the integration of an off-the-shelf USFDA-approved pulse oximeter with the mobile phone.

When reaching the “Record pulse and oxygen saturation” question in the mPneumonia application (shown in figure below) perform the following steps.


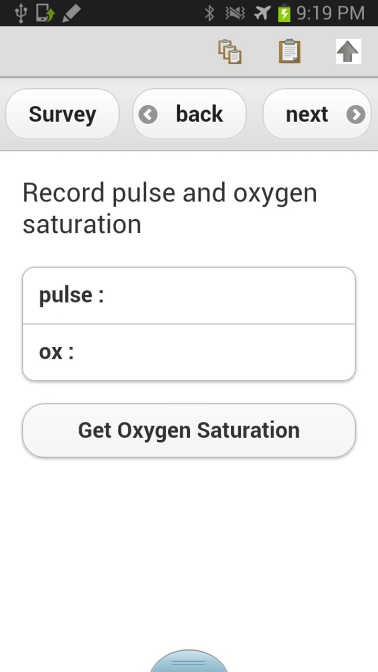


1. Verify the pulse oximeter is plugged into the USB port of the Android device. If pulse oximeter is not plugged into the Android device, plug the pulse oximeter into the USB port of the Android device (shown in the figure below). If this is the first time connecting the pulse oximeter please follow the instructions list in *Register Pulse Oximeter with ODK Sensors Framework and Android* section of the operating manual to register the pulse oximeter with the ODK Sensor Framework.


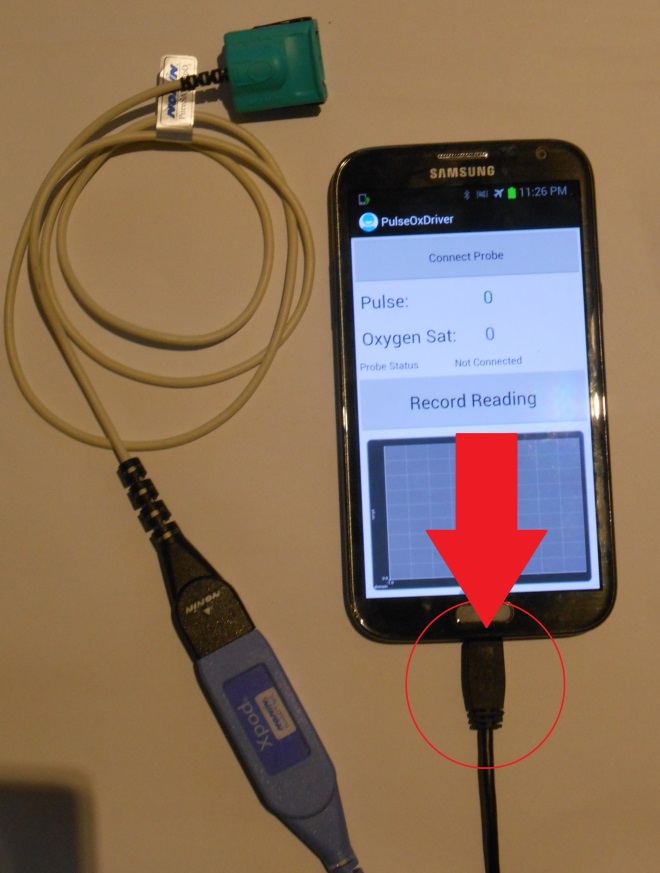


1. Click the “Get Oxygen Saturation” button (circled in red in the figure below).


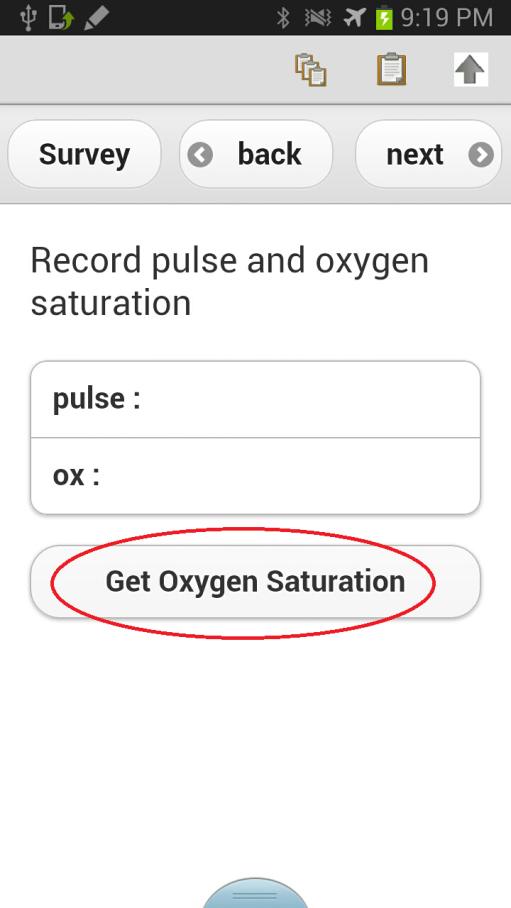


1. The pulse oximeter screen should appear (shown below in figure on the left) after pressing the ‘Get Oxygen Saturation’ button. NOTE: If the user recently plugged in the pulse oximeter a pop-up will appear from ODK Sensors asking permission to access the USB device (shown in figure below on the right). Click ‘OK’ on the pop-up to authorize ODK Sensors to access the pulse oximeter (this is a requirement of the Android operating system).


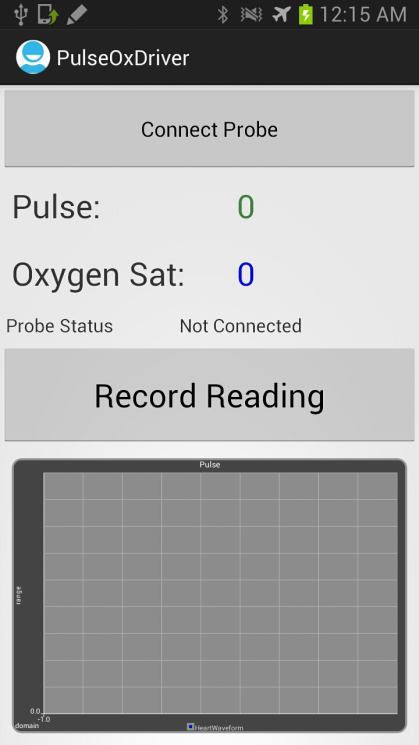

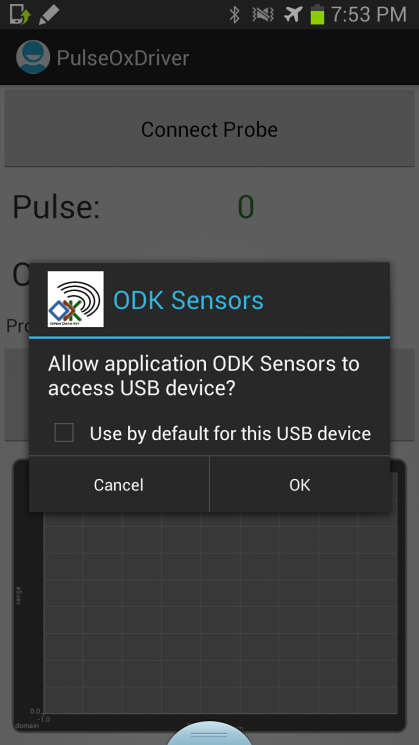


1. Place pulse oximeter sensor clip on the child’s finger. Use clean, dry finger where sensor will be placed. Gently squeeze the end of the sensor to open it and insert the finger all the way into the sensor. Make sure the finger symbol on the pulse oximeter sensor is on the top of the finger.


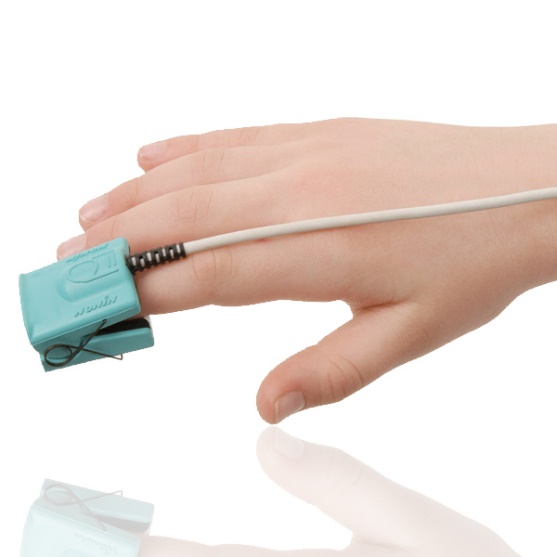


1. Next click the ‘Connect Probe’ button (circled in red in the figure below).


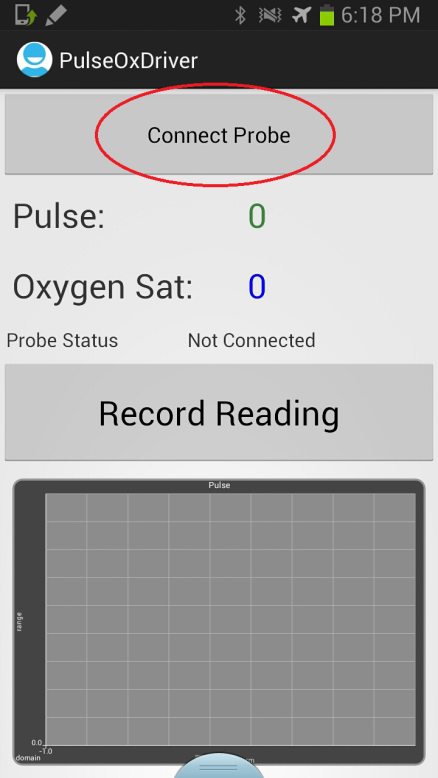


1. When the numbers are displayed in PINK it means the numbers are not stable and should not be trusted (NOTE: The probe status also reads “Inaccurate Data”). Initially the system MAY show “Error” in pink font while the Nonin Pulse Oximeter collects enough data to ensure an accurate reading (as shown in the figure below on the left). This “Error” data collection state may last for 30 seconds, if it lasts longer it is most likely a sign that a connection problem exists.
2. The system may also show some intermediate data that is inaccurate, which is denoted being inaccurate by being PINK and the probe status reads “Inaccurate Data” (as shown in the figure below on the right). If there is an error, check the child is not moving and the sensor is properly placed on a clean finger.


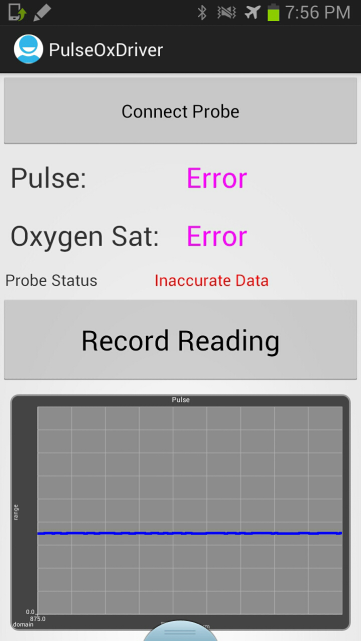

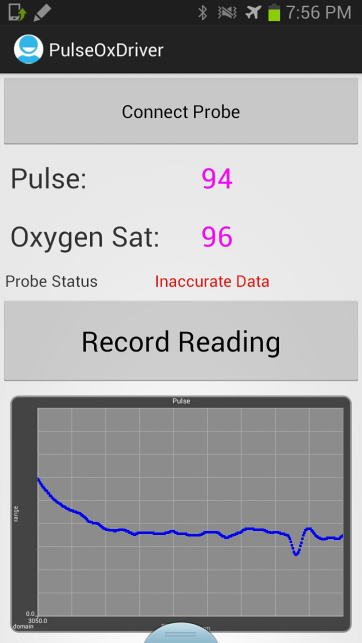


1. Once the probe status shows “Acceptable to Record” (additionally the pulse value text turns green and the oxygen saturation value text turns blue) press the ‘Record Reading’ button to record the pulse and oxygen saturation values into the IMCI protocol.


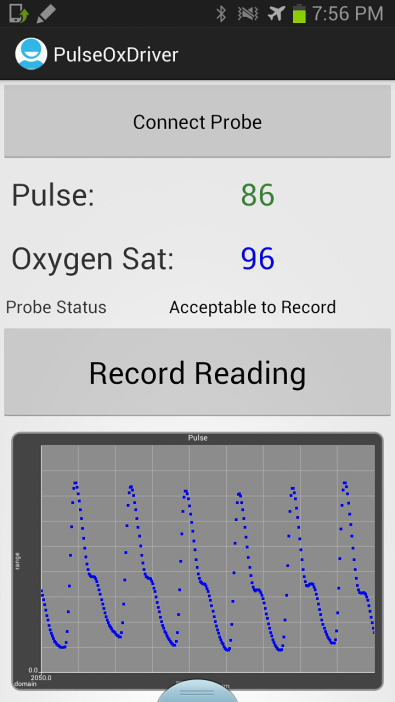

Supplement: S1 Manual — (DOCX) [file pone.0165201.s001.docx]
